# Aberrant Calreticulin Expression in Articular Cartilage of Dio2 Deficient Mice

**DOI:** 10.1371/journal.pone.0154999

**Published:** 2016-05-10

**Authors:** Nils Bomer, Frederique M. F. Cornelis, Yolande F. M. Ramos, Wouter den Hollander, Nico Lakenberg, Ruud van der Breggen, Lies Storms, P. Eline Slagboom, Rik J. U. Lories, Ingrid Meulenbelt

**Affiliations:** 1 Department of Molecular Epidemiology, LUMC, Leiden, Netherlands; 2 Laboratory of Tissue Homeostasis and Disease, Skeletal Biology and Engineering Research Centre, KU Leuven, Leuven, Belgium; 3 Division of Rheumatology, University Hospitals Leuven, Leuven, Belgium; University of Massachusetts Medical, UNITED STATES

## Abstract

**Objective:**

To identify intrinsic differences in cartilage gene expression profiles between wild-type- and *Dio2*^*-/-*^-mice, as a mechanism to investigate factors that contribute to prolonged healthy tissue homeostasis.

**Methods:**

Previously generated microarray-data (Illumina MouseWG-6 v2) of knee cartilage of wild-type and *Dio2* ^-/-^ -mice were re-analyzed to identify differential expressed genes independent of mechanical loading conditions by forced treadmill-running. RT-qPCR and western blot analyses of overexpression and knockdown of *Calr* in mouse chondro-progenitor cells (ATDC5) were applied to assess the direct effect of differential *Calr* expression on cartilage deposition.

**Results:**

Differential expression analyses of articular cartilage of *Dio2*^-/-^ (N = 9) and wild-type-mice (N = 11) while applying a cutoff threshold (P < 0.05 (FDR) and FC > |1,5|) resulted in 1 probe located in Calreticulin (*Calr*) that was found significantly downregulated in *Dio2*^-/-^ mice (FC = -1.731; *P* = 0.044). Furthermore, overexpression of *Calr* during early chondrogenesis in ATDC5 cells leads to decreased proteoglycan deposition and corresponding lower Aggrecan expression, whereas knocking down *Calr* expression does not lead to histological differences of matrix composition.

**Conclusion:**

We here demonstrate that the beneficial homeostatic state of articular cartilage in *Dio2*^*-/-*^ mice is accompanied with significant lower expression of *Calr*. Functional analyses further showed that upregulation of *Calr* expression could act as an initiator of cartilage destruction. The consistent association between *Calr* and *Dio2* expression suggests that enhanced expression of these genes facilitate detrimental effects on cartilage integrity.

## Introduction

Osteoarthritis (OA) is a prevalent, complex, chronic and disabling disease among elderly and is characterized by progressive destruction of joint cartilage, remodeling of the subchondral bone, formation of osteophytes and synovitis [1,2]. The aetiology of OA is not entirely understood, yet multiple factors such as joint injury, obesity and mechanical stress have been found to significantly contribute to the onset and progression of the disease. Moreover, OA has a considerable genetic component and a variety of genetic studies have identified multiple genes that robustly confer risk to OA [3,4]. A notable OA risk gene is the deiodinase iodothyronine type-2 (D2) gene (*DIO2*) [5] that has multiple lines of substantiating functional follow up data indicating that risk allele carriers have a more vivid upregulation of *DIO2* expression in articular cartilage. *In vitro* cell studies as well as a cartilage-specific transgenic rat study indicated that this resulted in cartilage destruction. In this *in vitro* model, inhibiting *DIO2* action, resulted in prolonged cartilage homeostasis, as where *Dio2*^*-/-*^-mice were shown to be protected against treadmill-running induced OA [6].Moreover, genome wide expression analysis of articular cartilage of *Dio2*^*-/-*^ and wild-type mice, before and after forced treadmill-running, demonstrated that particularly *Calr*, *Hmgb2*, *Sox4 and Socs2* were differentially regulated in the *Dio2*^*-/-*^ mice upon the mechanical applied stresses [6]. As *Dio2*^*-/-*^ mice had less cartilage damage these genes likely contribute to enhanced adaptive capacity to environmental challenges such as mechanical overloading. In parallel to these finding, there is need for insight into intrinsic molecular pathways that assure articular cartilage tissue homeostasis independent of environmental challenges. In the current study, we therefore re-analysed our micro-array expression data of wild type and *Dio2*^-/-^ mice independent of mechanical loading by treadmill-running and performed functional studies to validate results.

## Materials and Methods

### *DIO2*^-/-^ treadmill-running animal model and microarray assay

#### Animal experiments

As described previously [6], *Dio2*^-/-^-mice were a kind gift of Dr. V. Galton (Dartmouth Medical School, NH, USA) and were backcrossed onto the C57Bl/6 background. In the experiments reported here, mice were in the 6^th^ generation of backcrossing. Wild-type C57/Bl6 mice were purchased from Janvier (Le Genest St Isle, France). All experiments were approved by the Ethics Committee for Animal Research (KU Leuven, Belgium).

Four to 6-months old male *Dio2*^-/-^-and wild-type-mice were subjected to a forced treadmill-running regimen on a four lane modular treadmill (Columbus Instruments, Columbus, OH, USA). These mice ran for 3 weeks 1 hour/day, 5 days/week, at a speed of 11 m/min and with an inclination of 5°. All animals, including mice that were used as non-running controls, were littermates and were caged together and housed in the same facility [6]. Mice were group housed in standard mouse cages with sawdust as bedding material and under conventional laboratory conditions; constant room temperature (22 +/- 2°C), humidity level (55 +/- 5%), a 12-h light:12-h dark cycle (lights on at 8 AM) and standard food (Sniff, Soest, Germany) and water available ad libitum. Mice were euthanized by decapitation under sedation.

#### RNA isolation of murine knee cartilage

For gene expression studies, cartilage was micro-dissected, snap frozen in liquid nitrogen, and stored at -80° Celsius upon isolation. Cartilage of left-side knee-joints of *Dio2*^-/—^ (n = 6 NoRun and n = 12 Run) and wild-type-mice (n = 6 NoRun and n = 16 Run) was used for RNA isolation. To gain isolation efficiency and thereby mRNA quality, articular cartilage of knee-joints of two mice from the same sub-group were pooled resulting in n = 9 *Dio2*^*-/-*^ and n = 11 wild-type samples, analyzed in the manuscript [6].

#### Microarray analysis

Sample homogenization, RNA isolations, complementary DNA synthesis, amplification, biotin labeling and hybridization onto the Illumina MouseWG-6 v2 BeadChip microarrays (Illumina, Eindhoven, The Netherlands) were performed as described [6]. Samples were dispersed for experimental condition over the arrays of 6 chips from a single batch, and each chip contained a replicate sample that was also present on a second chip, to exclude batch effects across chips. Slide scanning, basic quality control and data normalization were performed as described [6]. Replicates measured over the different chips were taken into account when performing normalization. For further analysis, the replicate with the lowest number of calls was discarded from the dataset. Raw probe-level data were exported for analyses using the Limma R-package [7]. We checked the data for large-scale batch effects between chips using principal component analysis. Probes with P-value ≤0.05 after FDR correction for multiple testing were considered significant.

### Gain and loss of function of *Calr* in ATDC5, chondrogenic induction and histological evaluation

The murine chondrogenic cell line ATDC5 (kindly provided by H.C.M. Sips, LUMC) was maintained in DMEM/Ham’s F-12 (1:1 Gibco) supplemented with 5% FBS and antibiotics at 37°C in a humidified 10% CO2 / 90% atmosphere. To induce chondrogenesis, ATDC5 cells were plated in the medium described above supplemented with ITS+ (6.25 μg/ml insulin, 6.25 μg/ml transferrine, 6.25 ng/ml sodium selenite, 5.33 linoleic acid, and 1.25 μg/ml bovine serum albumine; Becton&Dickinson, Breda, The Netherlands). The medium was replaced every other day. The total cell culture time was 4 days.

For *Calr* overexpression purposes, a plasmid vector containing untagged mouse *Calr* (pCMV3-*mCalr*) was purchased (Sino Biological Inc., Beijing, P.R. China). As a control for the *Calr* overexpression experiments, ATDC5 cells were transfected with an empty pCMV3 vector. For knockdown of *Calr* expression, 3 unique 27mer siRNA duplexes, specific for *Calr*, were purchased (OriGene technologies, Rockville, MD, USA). Together with the 27mer siRNA duplexes a universal scrambled negative control siRNA duplex (OriGene technologies) was purchased to be used as a control for the knockdown experiments. The day before transfection, ATDC5 cells were passaged 1:6 from a confluent 6-well plate into a new 12-wells plate. Fugene® 6 transfection reagent was used to transfect ATDC5 cells with either the *mCalr*-vector or control vector, or the mixed siRNA duplexes or control duplex, according to the manufacturers protocol. One day after transfection, regular proliferation medium was changed for chondrogenesis medium. After 4 days of chondrogenesis, the cells were harvested for RNA and histology.

ATDC5 cells were fixed in 4% formaldehyde and stained for acidic polysaccharides (glycosaminoglycans (GAGs)) with Alcian Blue (8-GX pH = 1; Sigma-Aldrich). To measure the staining intensity, the Alcian Blue staining was washed with 6M guanidine hydrochloride (Sigma-Aldrich) to decolorize the extracellular matrix. Intensity of the supernatant was measured using a photospectrometer at 620 nm wavelength. Experiments were perfomed with biological triplo’s.

### *DIO2* overexpressing hBMSCs, 3D chondrogenic induction and mRNA evaluation

Previously, we performed *in vitro* 3D chondrogenesis using lentiviral DIO2 transduced hBMSCs[8]. Chondrogenesis was initiated in 0.5 ml serum-free chondrogenic differentiation medium (DMEM, supplemented with Ascorbic acid (50 μg/ml; Sigma-Aldrich; Zwijndrecht, The Netherlands), L-Proline (40 μg/ml; Sigma-Aldrich), Sodium Puryvate (100 μg/ml; Sigma-Aldrich), Dexamethasone (0,1 μM; Sigma-Aldrich), ITS+, antibiotics, and TGF-β1 (10 ng/ml; PeproTech)). Lentiviral constructs containing C-terminal FLAG-tagged cys-D2 (kindly provided by Prof. Dr. Bianco[9]) were constructed and transduced into hBMSCs as described before [8]. Here we used 3D pellets that were snap frozen in liquid nitrogen and stored at -80 degrees Celsius.

#### RNA isolation

RNA from the ATDC5 cells was isolated by lysing the cells in 500 μl of TRIzol® reagent (Life Technologies, Bleiswijk, The Netherlands), and 200 μl of chloroform was added before centrifugation (15 min at 14.000g). After addition of 1 v/v 70% ethanol/DEPC-treated water to the aqueous upper layer total RNA was isolated using Qiagen RNeasy mini columns following the manufacturer’s protocol. RNA isolation from the hBMSC pellets was performed by pooling two pellets for every given condition, respectively. The isolation of RNA was performed as described previously[10]. RNA quantity was assessed using a nanodrop spectrophotometer (Thermo Fisher Scientific Inc., Wilmington, USA).

#### Quantitative RT-PCR assay (validation)

Isolation of RNA was performed as described previously[6]. Approximately, 500 ng of total RNA was processed with the “First Strand cDNA Synthesis Kit” according to the manufacturer’s protocol (Roche Applied Science, Almere, The Netherlands), upon which cDNA was diluted 5 times. RT-qPCR measurements were performed on the Roche Lightcycler 480 II, using Fast Start Sybr Green Master reaction mix according to the manufacturer’s protocol (Roche Applied Science). Relative gene expressions of the Roche Lightcycler 480 II data were calculated by using the 2^-ΔΔCt^ method[11]. The housekeeping gene, glyceraldehyde 3-phosphate dehydrogenase (GAPDH) was used as a single reference gene for qPCR[12,13]. Primer efficiencies were verified by performing a concentration curve experiment (primer sequences used are listed in **S1 Table**). The student T-test was used to calculate the significance. All P-values < 0.05 were considered statistically significant.

## Results

**Fig 1:**
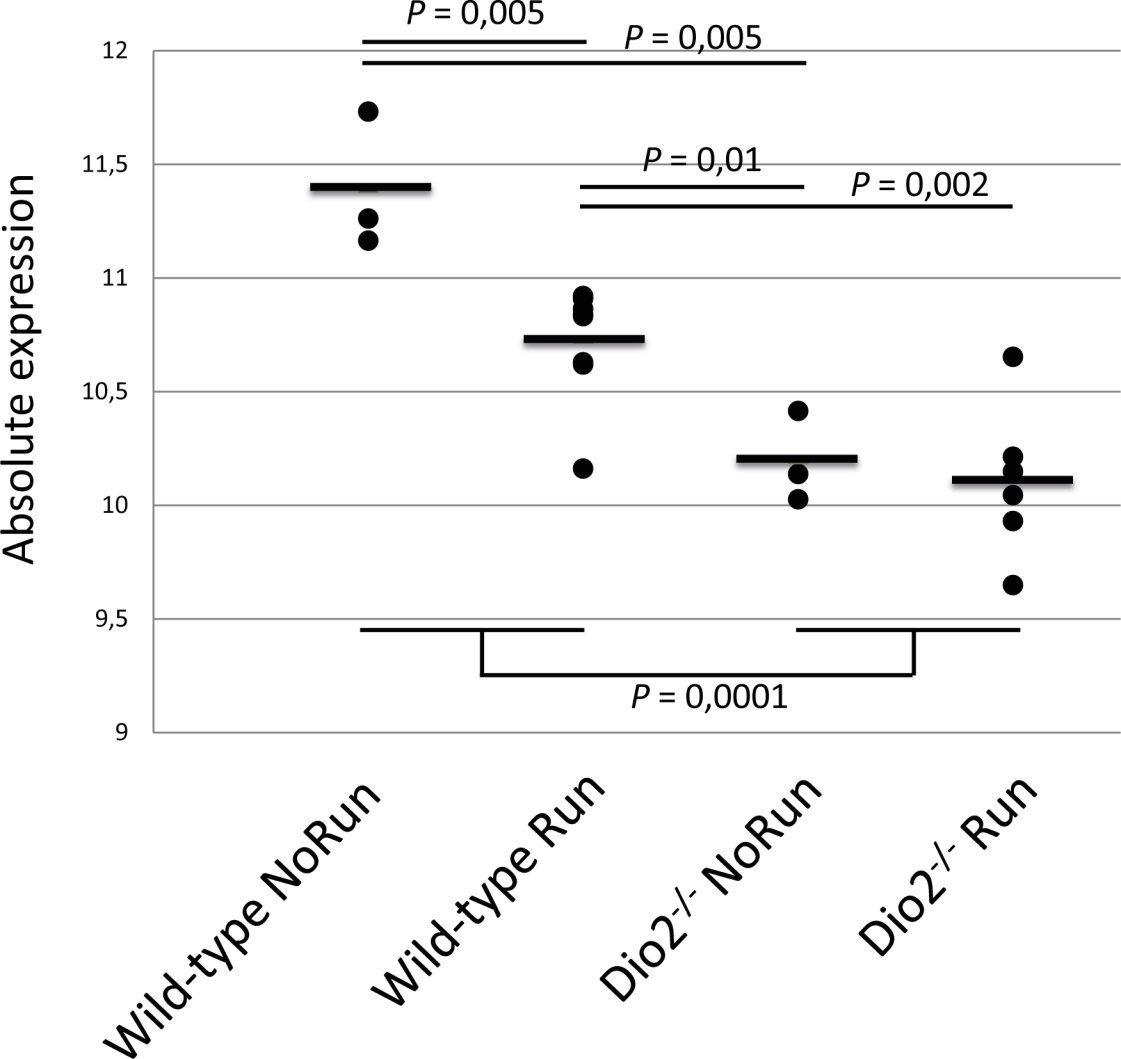
Calreticulin expression stratified for treadmill-running and knockout. Representation of the *Calr* expression values of all individual samples plotted by genotype-group (Wild-type and *Dio2*^*-/-*^) and treadmill-running-group (No Run and Run). Showing the significant reduction of *Calr* expression upon forced treadmill-running in wild-type-mice (*P* = 0,005) and the absence of change in the knockout-mice. P-values depicted are derived from Student T-Test.

After quality control and normalization of micro array expression data of articular cartilage of N = 9 *Dio2*^*-/-*^ and N = 11 wild-type mice, 18226 of the 45281 probes were found to have a detectable expression level, representing 12312 unique genes (~49% of the mouse transcriptome) and 2707 RIKEN sequences (**S2 Table**). Differential expression analyses while applying a cutoff threshold (P < 0.05 (FDR) and FC > |1,5|)[6] resulted in only 1 probe located in Calreticulin (*Calr*) that was found significantly downregulated in *Dio2*^-/-^ mice (FC = -1.731; *P* = 0.044). Technical validation by RT-qPCR in N = 20 samples (N = 9 *Dio2*^-/-^ and N = 11 wild type mice) of the discovery cohort confirmed the expression difference (**S1 Fig**). Notably, our previous data showed that forced treadmill-running had a profound effect on *Calr* expression (FC = -1,58, *P* = 0,0418)[6] in cartilage of wild-type-mice. However, stratifying expression of *Calr* by both mice type (Dio2^-/-^, Wildtype) and exposure (Run, Norun) shows that the here identified *Calr*-effect appears to be independent of the mechanical challenge (**Fig 1**).

**Fig 2:**
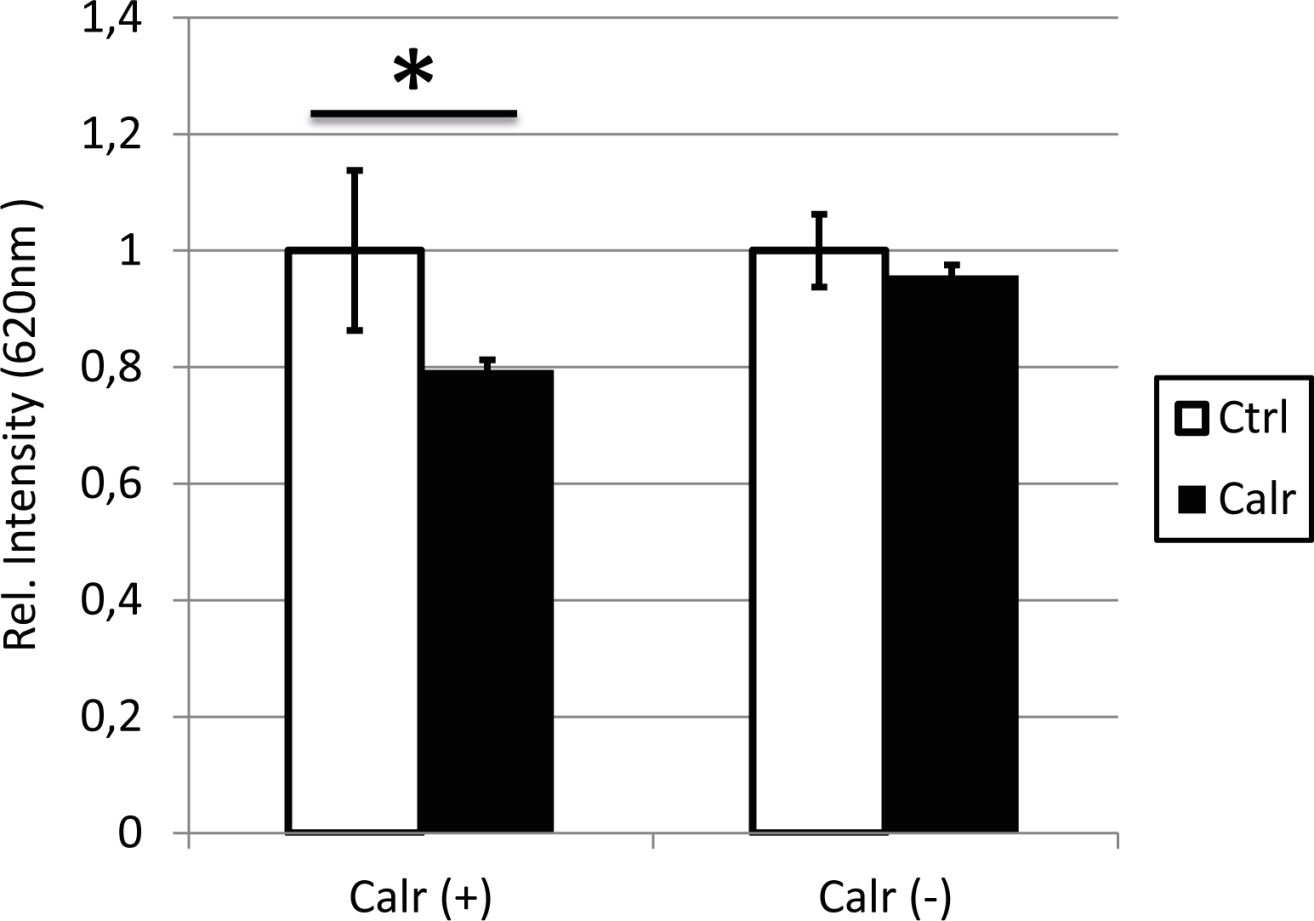
Overexpressing *Calr* resulted in significantly lower Alcian Blue (AB) staining intensities. Staining intensities of Alcian Blue staining measured with a photospectrometer (620 nm). Values are displayed as the average±SEM, relative to the control sample. Differences were analyzed with Student T-Test ((*) P < 0.05).

**Fig 3:**
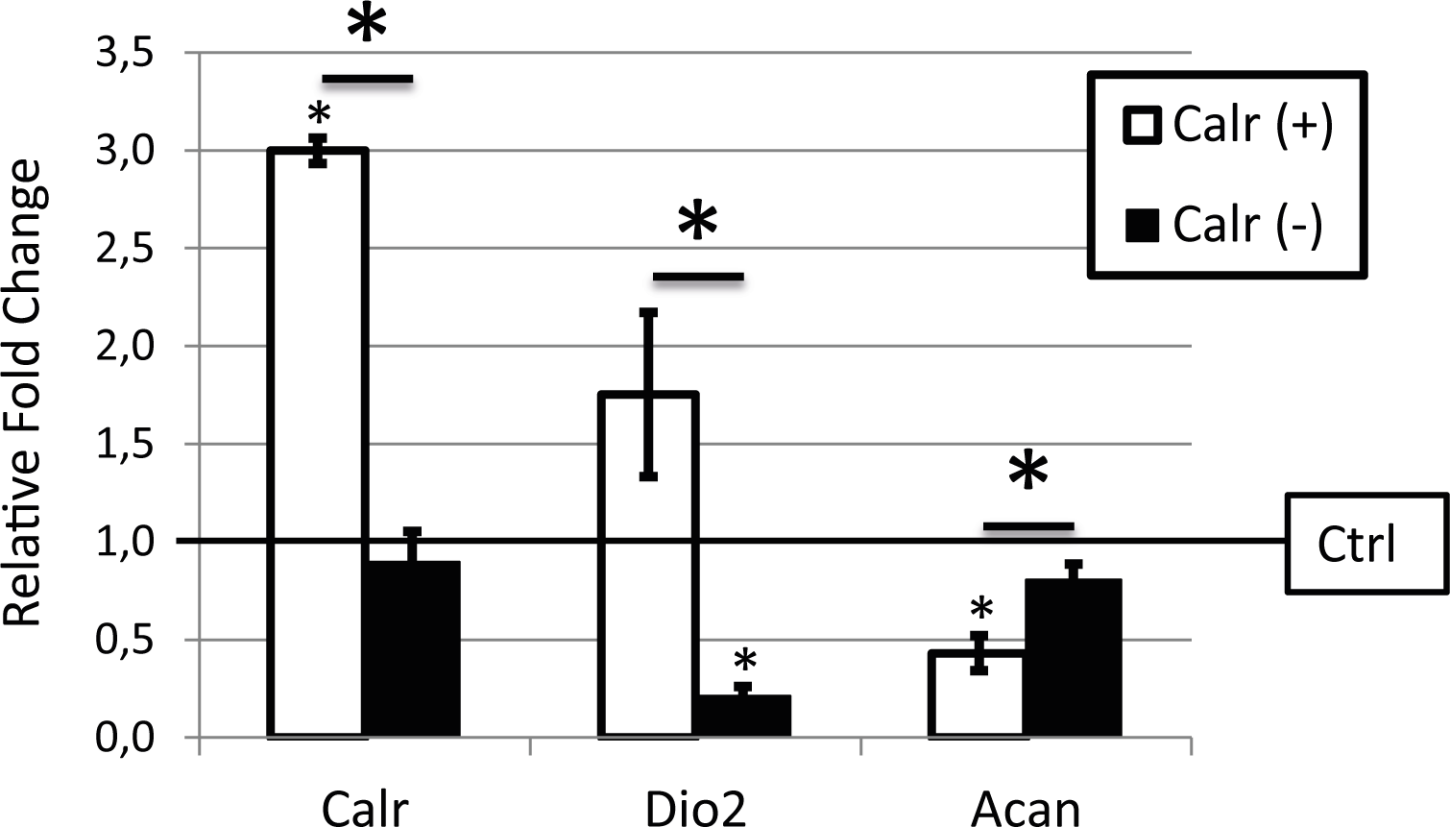
Expression analyses in *Calr* overexpressing ATDC5 cells. RT-qPCR expression of *Calr*, *Dio2 and Acan* in ATDC5 cells transfected with either a *Calr*-containing vector for overexpression (*Calr*+) or 3 unique 27mer siRNA duplexes, specific for Calr, for knockdown (*Calr*-). Values of the RT-qPCR are displayed as the average±SEM, normalized for *GAPDH* expression and relative to the control samples (dashed line). Differences were analyzed with Student T-Test ((*) P < 0.05).

Next, we investigated the effect of altered *Calr* expression on the chondrogenic capacity of ATDC5 cells. As measured by photospectrometry, overexpressing *Calr* during early 2D chondrogenesis resulted in significant lower Alcian Blue staining, indicating less glycosaminoglycan deposition (**Fig 2**). Concurrently, RT-qPCR confirmed significant lower mRNA expression levels of *Acan* (FC = -2,32) (**Fig 3**). Of note was the 1.8-fold upregulation of *Dio2* in the cells overexpressing *Calr*, albeit that this effect by definition was not significant (*P* = 0.08). On the other hand, knockdown of *Calr*, by siRNA duplexes in this model, did not show an effect on the amount of deposited glycosaminoglycans stained by Alcian Blue (**Fig 2**) nor on the mRNA expression levels of *Acan* (**Fig 3**). However, a significant 4.7-fold downregulated effect was observed for *Dio2* mRNA expression levels (*P* = 0,017).

**Fig 4:**
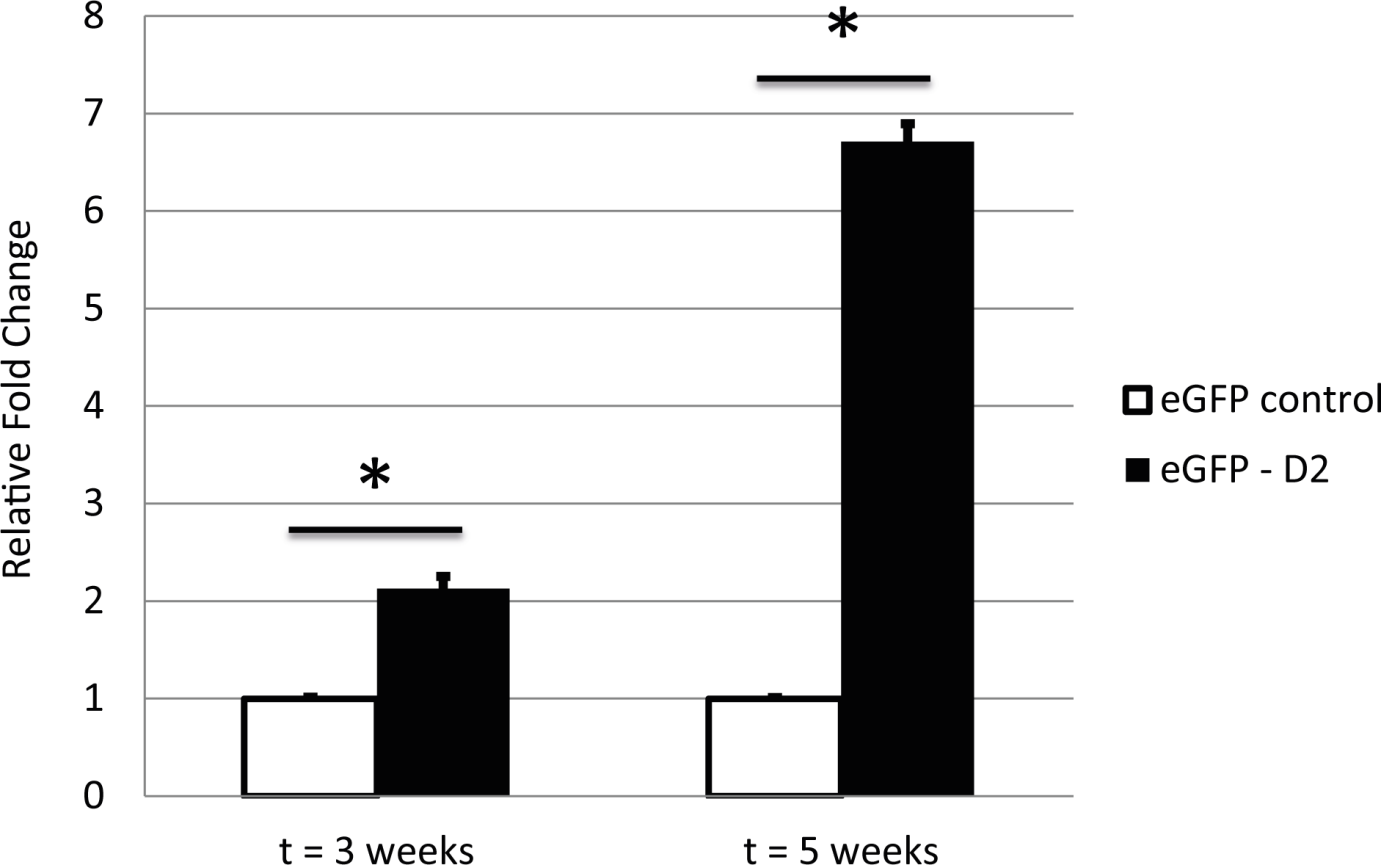
Calreticulin expression in *DIO2* overexpressing hBMSCs. qPCR expression of *Calr* in hBMSCs transduced with either a control virus-vector (eGFP) or a *DIO2*-eGFP vector. Values of the RT-qPCR are displayed as the average±SEM, normalized for *GAPDH* expression and relative to the control sample at every timepoint. Differences were analyzed with Student T-Test ((*) P < 0.05).

Finally, to confirm the transcriptional link between of *DIO2* and *CALR* expression, we examined *CALR* expression in cartilage constructs previously generated from human BMSCs *in vitro* 3D chondrogenesis and compared them to those generated while applying overexpression of *DIO2* by lentiviral transduction[8]. As shown in **Fig 4,** also in this human BMSCs *in vitro* 3D chondrogenesis model overexpressing *DIO2* coincided with significant higher *CALR* expression.

## Discussion

In parallel to disease modifiers, there is a need for insight into intrinsic factors that enhance stability of healthy articular chondrocytes. We show that cartilage of the *Dio2*^*-/-*^ as compared to wild type mice has an intrinsic difference in *Calr* expression. Since *Dio2*^*-/-*^ mice were previously found to be less susceptible to mechanically induced damage, our data indicate that mitigated *Calr* and/or *Dio2* expression, mark a beneficial homeostatic state of articular cartilage. Despite the, here outlined, consistent association between *Calr* and *Dio2* expression, no thyroid responsive elements were identified near *Calr*[14] nor did *Calr* show any predicted interactivity with thyroid hormone signaling based on String-DB [15].

*Calr* is a multifunctional protein that was shown to have many putative roles. Some examples are: Ca^2+^-binding, storage and stress induced release[16], quality control molecular chaperone binding to miss folded proteins and preventing them from being exported from the endoplasmic reticulum to the Golgi apparatus, and modulator of steroid-sensitive gene expression[17–21]. For that matter, the amino terminus of *Calr* interacts with the DNA-binding domain of nuclear hormone receptors (the glucocorticoid receptor[17], androgen receptor[18], and retinoic acid receptor[22]) and prevents the receptors from binding to their specific response elements, influencing transcriptional activities *in vitro* and *in vivo*. Furthermore, *Calr* expression was shown to be associated with cartilage thinning of mandibular cartilage in a rat model that studied the effects of compressive mechanical loading[16], and has been implicated in the pathogenesis of rheumatoid arthritis (RA)[23].

Similarly, in this respect, we showed that overexpression of *Calr* during early chondrogenesis in ATDC5 cells leads to decreased proteoglycan deposition and corresponding lower Aggrecan expression, whereas knocking down *Calr* expression does not lead to histological differences of matrix composition. It was shown before that increasing levels of *Calr*, sensitized the cells to apoptosis during mechanical-induced endoplasmatic reticulum-stress, while decreasing *Calr* levels protected cells from apoptosis[24]. It was hypothesised that by regulating the amount of calcium stored in the ER, and therefore the amount that can be released to the cytosol to trigger downstream events, *Calr* can affect apoptotic outcomes[25]. Given our data, we hypothesize that lower levels of *Calr* expression, as seen in the *Dio2*^*-/-*^-mice, could results in a more subtle calcium-induced apoptotic signal upon stress, being favourable for the maintenance of cartilage tissue homeostasis. The beneficial effect of reduced calcium-levels for cartilage integrity is supported by a recent study which reported that calcium antagonists might be efficient in preventing progression of OA[26].

Together, we here demonstrate that the beneficial homeostatic state of articular cartilage in *Dio2*^*-/-*^ mice is accompanied with significant lower expression of *Calr*. Moreover, we showed that increased *Calr* expression could directly provoke cartilage destruction. Given their consistent association, we conclude that enhanced interactive expression of *Calr* and *Dio2* contributes to detrimental effects on cartilage integrity. The precise molecular mechanism of *Calr* in the maintenance of cartilage homeostasis and its interplay with *Dio2* signalling is, however, little explored and more research is therefore needed.

## Supporting Information

S1 Fig Expression validation of *Calr* by RT-qPCR. Values of the RT-qPCR are displayed as the average±SEM, normalized for *GAPDH* expression and relative to the control samples. Differences were analyzed with Student T-Test ((*) P < 0.05).
(TIF)

S1 Table Primer sequences used for RT-qPCR. (XLSX)

S2 Table Microarray data (FDR-corrected P-values) of probes with detectable expression levels. (XLSX)
