# Supplementary figures and images for: Aberrant Calreticulin Expression in Articular Cartilage of Dio2 Deficient Mice
Source: PLoS One. 2016 May 10;11(5):e0154999. doi: 10.1371/journal.pone.0154999 (PMC4862667; doi:10.1371/journal.pone.0154999)

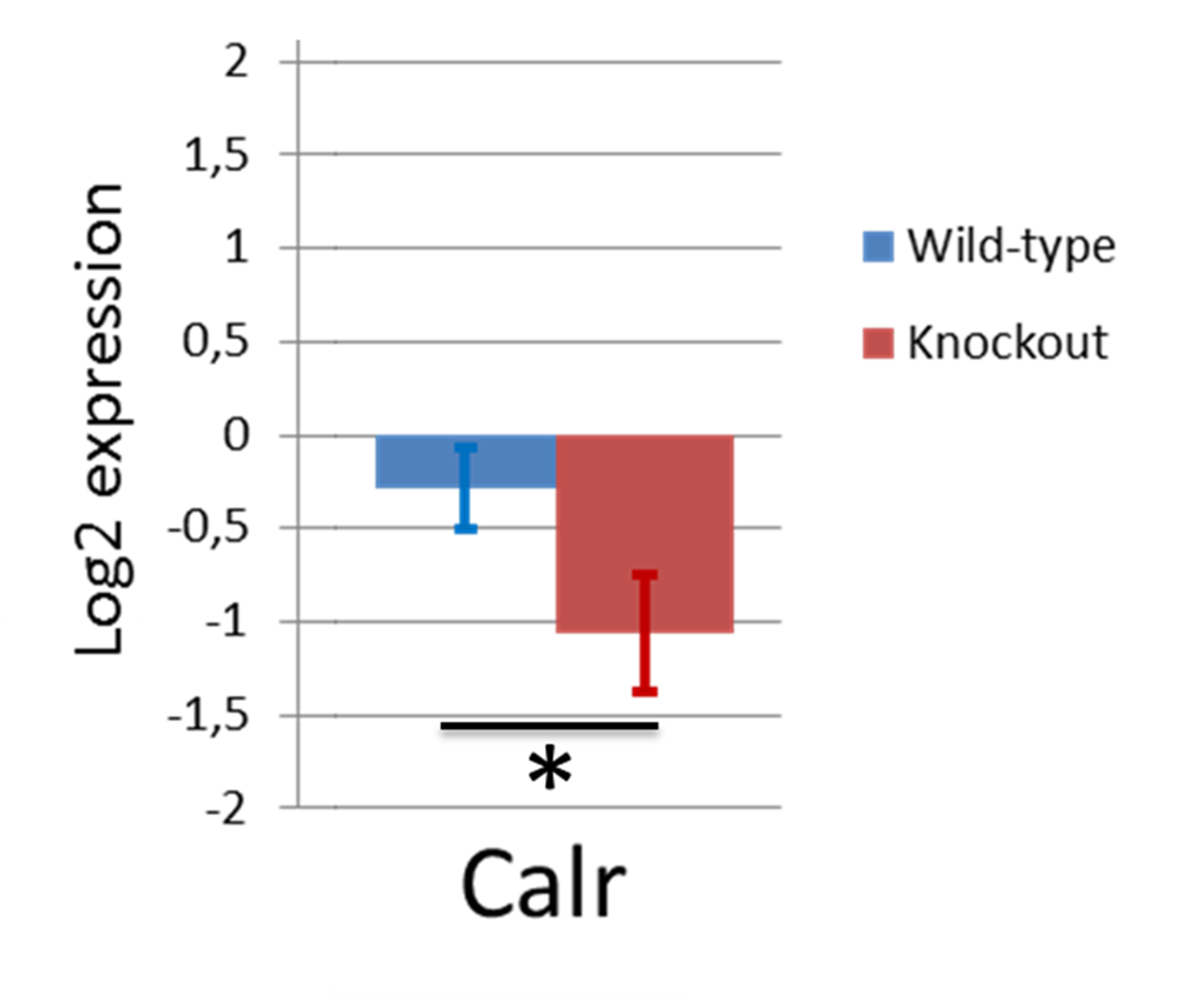

Supplement: S1 Fig — Values of the RT-qPCR are displayed as the average±SEM, normalized for GAPDH expression and relative to the control samples. Differences were analyzed with Student T-Test ((*) P < 0.05). (TIF) [file pone.0154999.s001.tif]
